# Concomitant limaprost use and intraocular pressure response to latanoprost in normal-tension glaucoma: A retrospective cohort study

**DOI:** 10.1097/MD.0000000000050838

**Published:** 2026-09-25

**Authors:** Heewon Bae, Tae Gu Kang

**Affiliations:** a Department of Neurology, Ilsan Paik Hospital, Inje University College of Medicine, Goyang, Republic of Korea; b Department of Ophthalmology, Yonsei Bom Eye Clinic, Gyeonggi-do, Republic of Korea.

**Keywords:** intraocular pressure, latanoprost, limaprost, normal-tension glaucoma, prostaglandin analogs

## Abstract

Normal-tension glaucoma (NTG) is a progressive optic neuropathy in which intraocular pressure (IOP) reduction is important for limiting disease progression. Latanoprost is commonly used as first-line therapy, but treatment response varies. We investigated whether concomitant oral limaprost use was associated with the IOP response to latanoprost in patients with NTG. We retrospectively reviewed patients with NTG treated between January 2015 and August 2021 at the Veterans Health Service Medical Center. The final matched cohort included 446 patients: 223 patients receiving latanoprost with concomitant limaprost and 223 age- and sex-matched controls receiving latanoprost alone. Three-month IOP was analyzed at the patient level.Multivariable linear regression was performed with 3-month IOP as the outcome, adjusting for baseline IOP, age, sex, nonsteroidal anti-inflammatory drug use, systemic beta-blocker use, diabetes, and hypertension. Baseline IOP was similar between groups. The median IOP reduction at 3 months was 1.0 mm Hg [interquartile range (IQR), −0.5 to 2.5] in the limaprost group and 2.0 mm Hg [IQR, 0.5–3.0] in controls. In the adjusted analysis, concomitant limaprost use was associated with a 0.91-mm Hg higher IOP at 3 months (95% confidence interval, 0.54 to 1.27; *P* < .001). In a secondary exploratory subgroup of 63 patients who subsequently received add-on dorzolamide/timolol, 35 were in the limaprost group and 28 were controls; no statistically significant between-group difference in IOP reduction was detected. Concomitant limaprost use was associated with a modestly smaller observed IOP reduction after latanoprost treatment in patients with NTG. Because of the retrospective design and the modest magnitude of the association, prospective studies are needed to determine whether a clinically meaningful pharmacologic interaction exists.

**What is already known on this topic**

-  Latanoprost is a commonly used first-line prostaglandin analog for lowering intraocular pressure in open-angle glaucoma.

-  The IOP response to prostaglandin analogs varies among patients, and concomitant systemic medications may contribute to this variability.

**What this study adds**

- Concomitant limaprost use was associated with a smaller observed IOP reduction after latanoprost treatment in this retrospective matched cohort.
- The association persisted after adjustment for baseline IOP and relevant systemic covariates.

**How this study might affect research, practice or policy**

- The findings support prospective evaluation of a possible interaction between systemic limaprost use and the IOP response to latanoprost.
- The present observational data do not by themselves support changing glaucoma therapy.

## 1. Introduction

Glaucoma is a progressive optic neuropathy characterized by degeneration of the retinal ganglion cells and retinal nerve fiber layers (RNFL) that results in changes in the optic nerve head. It is the leading cause of irreversible visual impairment and blindness worldwide.^[1]^ The most common form of glaucoma is open-angle glaucoma. Open-angle glaucoma can be classified into primary open-angle glaucoma and normal-tension glaucoma (NTG), which have similar structural and functional impairment.^[2]^ NTG constitutes the majority of open-angle glaucoma cases in Asians.^[3]^The proportion of NTG, which needs careful surveillance, is significantly higher in the Asian population than in other people. Intraocular pressure (IOP) levels influence the pathogenesis of NTG as they are associated with optic-nerve ischemia. Therefore, lowering IOP is beneficial in preventing disease progression, including visual field (VF) loss or reduction in the RNFL thickness.^[4]^

Latanoprost is widely used as a first-line therapy for lowering IOP in patients with glaucoma.^[5]^It is a prostaglandin F2α (PGF2α) analog that lowers IOP by an average of 35%. However, some patients do not respond to latanoprost (nonresponder), and IOP does not decrease adequately after therapy. Previous studies have reported that a subset of patients show limited IOP reduction with latanoprost.^[6]^Although the reason for the failure of IOP reduction remains to be clarified, we should consider the possibility that glaucoma control may be affected by other drugs.Limaprost is a prostaglandin E1 (PGE1) derivative used to treat intermittent neurogenic claudication in patients with lumbar spinal stenosis.^[7]^ Limaprost is distinct from nonsteroidal anti-inflammatory drugs (NSAIDs), although the 2 classes may be prescribed concomitantly in patients with spinal stenosis.^[8,9]^PGE1 derivatives have been reported to improve blood flow and neurologic function in experimental models of neural tissue ischemia.^[10]^Because limaprost and latanoprost act through different prostanoid pathways, a pharmacologic interaction is biologically conceivable; however, such an interaction remains hypothetical.

To the best of our knowledge, the association between concomitant limaprost use and the IOP response to latanoprost has not been well characterized. We therefore investigated whether concomitant limaprost use was associated with a smaller IOP reduction after latanoprost treatment in patients with NTG.

## 2. Methods

### 2.1. Data source and study population

We reviewed the medical records of 2819 patients diagnosed with normal-tension glaucoma (NTG) between January 2015 and August 2021 at the Veterans Health Service (VHS) Medical Center. Among these patients, 1026 had been prescribed both latanoprost and limaprost, whereas 1793 had been prescribed latanoprost without limaprost.

Among the 1026 patients who had received both medications, 635 patients in whom latanoprost had been initiated before limaprost were excluded, leaving 391 potentially eligible patients. Of these, 93 patients were excluded because of loss to follow-up, 52 because follow-up IOP measurements were unavailable, and 23 because glaucoma medication was changed owing to latanoprost-related adverse effects. Consequently, 223 patients were included in the concomitant limaprost group.

Among the 1524 patients receiving latanoprost without limaprost who had available follow-up IOP data, 223 controls were selected using 1:1 nearest-neighbor matching on age and sex without replacement. No prespecified age caliper was applied. Each control could be selected only once. The final matched cohort, therefore, comprised 446 patients: 223 patients receiving concomitant limaprost and 223 controls (Fig. 1).

**Figure 1.**
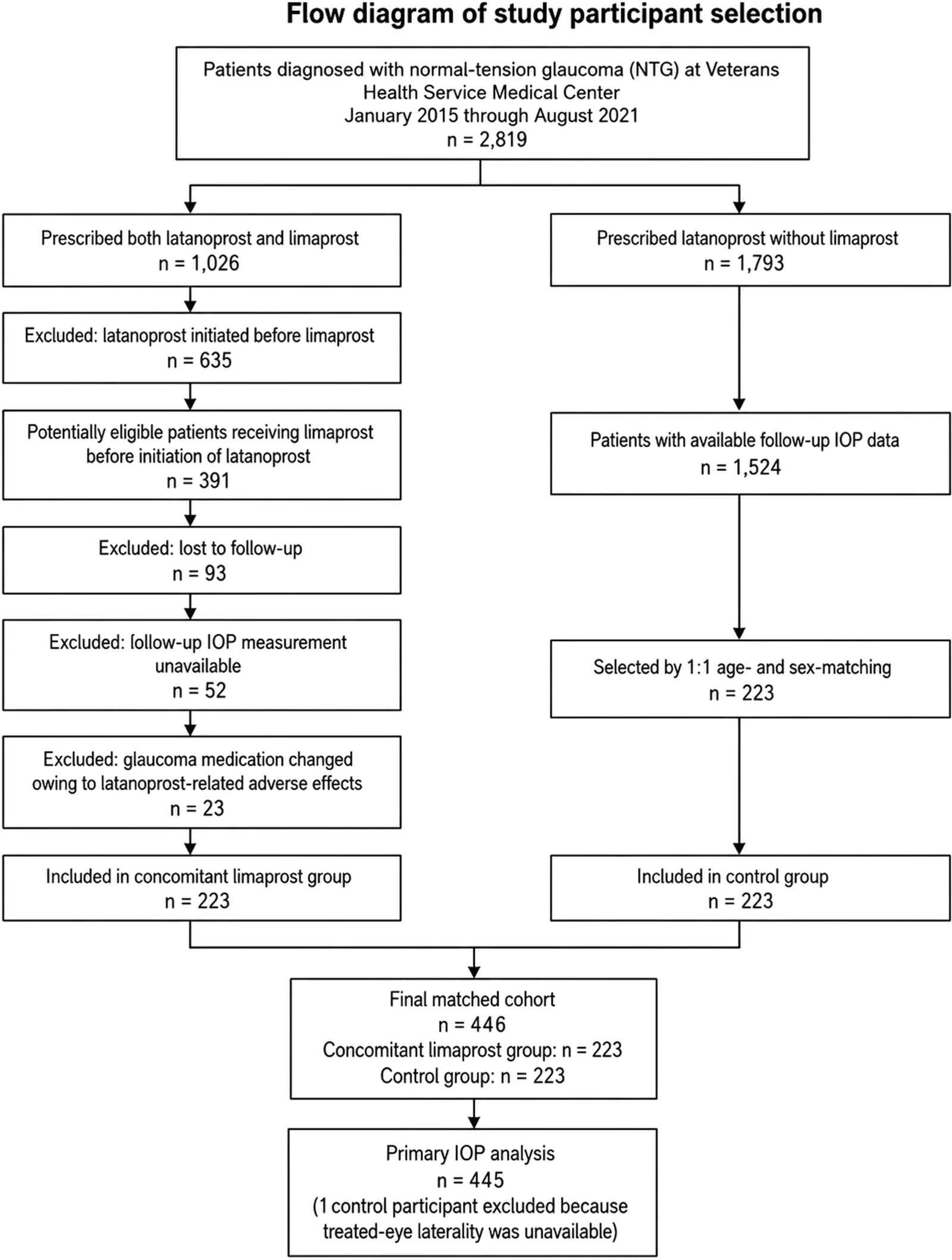
illustrates the selection of 446 normal-tension glaucoma patients: 223 received latanoprost with concomitant limaprost, and 223 age- and sex-matched controls received latanoprost alone.

The detailed inclusion and exclusion criteria were as follows:

- Inclusion
  - Patients with typical glaucomatous optic neuropathy with corresponding VF loss, open anterior-chamber angles, and IOP ≤ 21 mmHg
  - Patients who had been diagnosed but not treated
  - Patients who had been receiving limaprost for more than 6 weeks before the initiation of latanoprost.
- Exclusion
  - Patients with a history of glaucoma or use of topical antiglaucoma agents
  - Patients who had previous incisional ocular surgery in either eye
  - Patients who had ocular infection or inflammation within 6 months of enrollment.

nonresponse to latanoprost was defined as an IOP reduction of < 10% from baseline at the 3-month follow-up in patients with glaucomatous VF defects and RNFL damage. The VF and RNFL abnormalities represented baseline glaucomatous findings rather than documented progression during the 3-month follow-up. A total of 63 patients met this definition, including 35 patients in the concomitant limaprost group and 28 controls. These patients subsequently received add-on dorzolamide/timolol therapy and were included in the secondary analysis.The study followed the tenets of the Declaration of Helsinki and was approved by the Institutional Review Board of the Veterans Health Service Medical Center (IRB No. BOHUN 2022-12-012-001). The requirement for informed consent was waived because of the retrospective nature of the study and the use of de-identified data. Patient information was anonymized and de-identified before analysis.

### 2.2. Drug administration

Participants with NTG were prescribed 1 drop daily of 0.005% latanoprost (Xalatan®; 50 μg/mL) and were advised to instill the medication in the evening. IOP was reassessed approximately 3 months after latanoprost initiation. Patients in the secondary exploratory subgroup subsequently received dorzolamide/timolol (Cosopt®) twice daily. Limaprost was prescribed at 5 μg 3 times daily. Concomitant limaprost exposure was identified from medication records at the time latanoprost treatment was initiated.Concomitant limaprost exposure was defined as documented limaprost use for more than 6 weeks before the initiation of latanoprost. Exposure classification was based on medication records available at the time of latanoprost initiation; continuous limaprost use throughout the subsequent 3-month follow-up could not be reliably confirmed. Because this was a retrospective chart-review study, adherence to both limaprost and latanoprost throughout follow-up could not be reliably verified from the available records.

### 2.3. IOP measurements

IOP was measured using a noncontact tonometer (Topcon CT80 Computerized Tonometer; Topcon, Tokyo, Japan), and the average of three consecutive measurements was recorded. The unit of analysis was the patient. For patients receiving unilateral latanoprost treatment, the IOP of the treated eye was used. For patients receiving bilateral treatment, the mean IOP of both treated eyes was used. The same eye-selection rule was applied at baseline and at the 3-month follow-up so that each patient contributed a single IOP value at each time point. One control participant with unavailable treated-eye laterality was excluded from the primary IOP analysis. The primary outcome was 3-month IOP after initiation of latanoprost. The unadjusted change in IOP from baseline to 3 months was also summarized descriptively. The secondary exploratory outcome was the change in IOP after add-on dorzolamide/timolol therapy in the 63-patient add-on therapy subgroup.

### 2.4. Statistical analysis

Continuous variables were summarized as mean (standard deviation) or median (interquartile range), as appropriate, and categorical variables as number (percentage). Baseline characteristics were compared between the concomitant limaprost and control groups, and standardized mean differences were additionally reported to assess covariate balance after matching.

Unadjusted between-group differences in IOP change were assessed using the Mann–Whitney *U* test. The primary adjusted analysis used a patient-level multivariable linear regression model with 3-month IOP as the dependent variable, concomitant limaprost use as the primary exposure, and baseline IOP, age, sex, NSAID use, systemic beta-blocker use, diabetes, and hypertension as covariates.HC3 robust standard errors were used, and adjusted effect estimates are presented with 95% confidence intervals. A complete-case approach was used for the primary adjusted analysis, and no imputation was performed.One control participant without recorded treated-eye laterality was excluded from the primary IOP analysis, resulting in an analytic sample of 445 patients.

Although the cohort had originally been constructed using 1:1 age- and sex-matching, identifiers linking individual matched pairs were not retained in the final analytic dataset. Therefore, pair-specific covariance could not be incorporated directly into the regression model. Age and sex, the original matching variables, were included as covariates in the adjusted analysis.

As a sensitivity analysis, central corneal thickness and average RNFL thickness were additionally included as covariates in the primary multivariable model after excluding participants with missing or implausible values for these variables. HC3 robust standard errors were used as in the primary analysis.

## 3. Results

### 3.1. Demographic characteristics of the study population

The final matched cohort included 446 participants, with 223 patients in each group. Overall, 374 participants (83.9%) were men and 72 (16.1%) were women. Each group included 187 men and 36 women.Mean age was 74.5 years in the limaprost group and 74.0 years in controls. Baseline IOP was similar between groups. Hypertension and NSAID use were more frequent in the limaprost group, supporting the use of multivariable adjustment. Baseline characteristics and standardized mean differences are summarized in Table 1.

**Table 1:** Clinical characteristics of patients included in the study.

Characteristic	Treatment	Control	SMD
	(N = 223)	(N = 223)	
Age	74.5 (6.0)	74.0 (4.7)	0.091
Sex			0.000
Male	187 (83.9%)	187 (83.9%)	
Female	36 (16.1%)	36 (16.1%)	
Ophthalmic characteristics			
Central corneal thickness (μm)	535.1 (36.3)	536.9 (33.7)	0.053
Average RNFL thickness (μm) †	81.1 [70.0, 89.0]	77.3 [66.5, 87.5]	0.244
Baseline IOP (mm Hg)	15.0 (2.8)	15.3 (3.6)	0.094
Comorbidity			
Diabetes mellitus	73 (32.7%)	75 (33.6%)	0.019
Systemic hypertension	127 (57.0%)	103 (46.2%)	0.217
Concomitant systemic medications			
NSAID	47 (21.1%)	12 (5.4%)	0.476
Antidepressant	19 (8.5%)	17 (7.6%)	0.033
Levodopa	1 (0.4%)	1 (0.4%)	0.000
Anxiolytic	9 (4.0%)	9 (4.0%)	0.000
Systemic beta-blocker	28 (12.6%)	19 (8.5%)	0.133
Antihistamine	8 (3.6%)	7 (3.1%)	0.026

Data are presented as mean (SD), median [IQR], or n (%), as appropriate.

IOP = intraocular pressure, NSAID = nonsteroidal anti-inflammatory drug, RNFL = retinal nerve fiber layer, SMD = standardized mean difference.

### 3.2. Comparison of IOP in patients with or without limaprost

Baseline IOP did not differ significantly between groups. At 3 months, median IOP was 14.0 mm Hg [IQR, 12.0–16.0] in the limaprost group and 13.0 mm Hg [IQR, 11.5–15.0] in controls. Median IOP reduction was 1.0 mm Hg [IQR, −0.5 to 2.5] and 2.0 mm Hg [IQR, 0.5–3.0], respectively (*P* < .001). The primary IOP analysis included 445 of the 446 matched participants because one control participant lacked recorded treated-eye laterality. In the multivariable model adjusted for baseline IOP, age, sex, NSAID use, systemic beta-blocker use, diabetes, and hypertension, concomitant limaprost use was associated with a 0.91-mm Hg higher IOP at 3 months (adjusted β = 0.905 mm Hg; 95% CI, 0.541 to 1.268; *P* < .001) (Table 2).

**Table 2:** Comparison of intraocular pressure with or without limaprost.

	Treatment	Control	*P*-value
	(N = 223)	(N = 222)	
Baseline IOP (mm Hg)	15.0 [13.0, 17.0]	14.5 [12.5, 17.5]	.784
IOP after 3 months (mm Hg)	14.0 [12.0, 16.0]	13.0 [11.5, 15.0]	.001
IOP reduction (mm Hg)	1.0 [-0.5, 2.5]	2.0 [0.5, 3.0]	<.001
**Adjusted analyses**			
Primary multivariable model	**0.905 (0.541–1.268**)	**445**	**<.001**
Sensitivity model including CCT and RNFL	**1.022 (0.620–1.424**)	**336**	**<.001**

Values are presented as median [IQR].

CCT = central corneal thickness, CI = confidence interval, IOP = intraocular pressure, RNFL = retinal nerve fiber layer.

Unadjusted between-group comparisons were performed using the Mann–Whitney *U* test. The primary multivariable model adjusted for baseline IOP, age, sex, NSAID use, systemic beta-blocker use, diabetes, and hypertension. The sensitivity model additionally adjusted for central corneal thickness and average RNFL thickness after exclusion of participants with missing or implausible values. HC3 robust standard errors were used in both adjusted models.

In a sensitivity analysis additionally adjusting for central corneal thickness and average RNFL thickness, the association remained materially unchanged (adjusted β = 1.022 mm Hg; 95% CI, 0.620–1.424; *P* < .001; n = 336).

### 3.3. Characteristics of latanoprost non-responders

A total of 63 patients met the predefined criteria for nonresponse to latanoprost, defined as an IOP reduction of < 10% from baseline at the 3-month follow-up in patients with baseline glaucomatous VF defects and RNFL damage. Of these, 35 were in the concomitant limaprost group and 28 were controls. The limaprost subgroup included 29 men and 6 women, whereas the control subgroup included 25 men and 3 women. Baseline characteristics of the non-responders are shown in Table 3.

**Table 3:** Clinical characteristics of latanoprost non-responders unit: mean (SD), median [IQR], or n (%).

Characteristic	Treatment	Control
	(N = 35)	(N = 28)
Age, years	74.0 [72.0, 76.0]	71.5 [70.8, 74.0]
Sex		
Male	29 (82.9%)	25 (89.3%)
Female	6 (17.1%)	3 (10.7%)
Ophthalmic characteristics		
Central corneal thickness (μm)	543.8 (35.9); n = 30	532.8 (42.2); n = 26
Average RNFL thickness (μm)	74.0 [61.3, 84.8]; n = 35	71.0 [58.0, 75.0]; n = 27
Baseline IOP (mm Hg)	16.0 (3.2)	15.2 (3.2)
Comorbidity		
Diabetes mellitus	10 (28.6%)	11 (39.3%)
Systemic hypertension	20 (57.1%)	14 (50.0%)
Concomitant systemic medications		
NSAID	6 (17.1%)	0 (0%)
Antidepressant	4 (11.4%)	4 (14.3%)
Levodopa	0 (0%)	0 (0%)
Anxiolytic	4 (11.4%)	1 (3.6%)
Systemic beta-blocker	5 (14.3%)	1 (3.6%)
Antihistamine	1 (2.9%)	0 (0%)

Data are presented as mean (SD), median [IQR], or n (%), as appropriate. IOP = intraocular pressure, NSAID = nonsteroidal anti-inflammatory drug, RNFL = retinal nerve fiber layer.

### 3.4. IOP response after add-on dorzolamide/timolol

The 63 non-responders subsequently received add-on dorzolamide/timolol therapy. Median IOP reduction after add-on therapy was 3.0 mm Hg [IQR, 0.0–4.0] in the limaprost group and 2.0 mm Hg [IQR, 0.75–3.25] in controls. The between-group difference was not statistically significant (*P* = .379) (Table 4). Because this was a secondary analysis with a relatively small sample, the absence of a statistically significant difference should not be interpreted as evidence of equivalence or absence of an interaction.

**Table 4:** IOP response after add-on dorzolamide/timolol in latanoprost non-responders.

IOP changes	Treatment	Control	*P-value*
	(n = 35)	(n = 28)	
Before add-on therapy (mm Hg)	17.0 [13.5, 19.0]	14.5 [12.75, 17.0]	
After add-on therapy (mm Hg)	14.0 [13.0, 16.0]	14.0 [10.75, 15.0]	
IOP reduction (mm Hg)	3.0 [0.0, 4.0]	2.0 [0.75, 3.25]	0.379

Values are presented as median [IQR]. The between-group comparison of IOP reduction was performed using the Mann–Whitney *U* test.

Values are presented as median [IQR]. IOP = intraocular pressure. Between-group comparison of IOP reduction was performed using the Mann–Whitney *U* test.

## 4. Discussion

This study investigated the association between concomitant limaprost use and the IOP response to latanoprost in patients with NTG. Concomitant limaprost use was associated with a smaller observed IOP reduction after latanoprost treatment. This association persisted after adjustment for baseline IOP and relevant systemic covariates, with an adjusted between-group difference of approximately 0.9 mm Hg in 3-month IOP. Although statistically significant, the magnitude of this difference was modest, and its clinical significance should therefore be interpreted cautiously. In the small secondary subgroup receiving add-on dorzolamide/timolol, no statistically significant between-group difference in subsequent IOP reduction was detected.

Latanoprost is a PGF2α analog widely used as first-line therapy because of its ocular hypotensive efficacy and favorable safety profile.^[11]^ Previous studies have demonstrated interindividual variability in the response to prostaglandin analogs, including a subset of patients with limited IOP reduction.^[12,13]^ Concomitant systemic medications may also contribute to variability in IOP response.^[14]^ In particular, previous studies and a meta-analysis have suggested that NSAIDs may modify or augment the IOP-lowering effect of prostaglandin analogs.^[15,16]^ These observations support consideration of systemic medication use when evaluating treatment response, although they do not establish a specific pharmacologic interaction between limaprost and latanoprost.

Experimental studies have reported that cyclooxygenase inhibition may alter prostanoid receptor density, including EP and FP receptors.^[17]^ Prostaglandin analogs may also influence ciliary muscle tone and extracellular matrix remodeling, thereby facilitating aqueous humor outflow.^[18]^ Limaprost is a PGE1 derivative, whereas latanoprost primarily acts through the FP receptor pathway.^[9,19]^ One possible explanation for the observed association is an interaction involving prostanoid signaling or vascular dynamics within the ciliary body. However, these mechanisms remain speculative. The present study did not include receptor-level or ocular blood-flow measurements, and its retrospective observational design cannot establish a pharmacologic interaction between limaprost and latanoprost.

This study has several limitations. First, its retrospective design precludes causal inference and leaves open the possibility of residual confounding and confounding by indication. Patients receiving limaprost may differ systematically from controls because of spinal stenosis and other underlying health characteristics. Second, medication adherence and continuous limaprost exposure throughout the entire 3-month follow-up could not be reliably confirmed from the available records. Third, IOP measurements were not standardized to an identical time of day, and diurnal variability may have influenced individual measurements. Fourth, one control participant lacked information regarding treated-eye laterality and was excluded from the primary IOP analysis. Finally, the secondary add-on dorzolamide/timolol analysis involved a small, selected subgroup and may therefore be susceptible to selection bias, regression to the mean, and limited statistical power. Accordingly, the absence of a statistically significant between-group difference in this subgroup should not be interpreted as evidence of equivalence or absence of an interaction.

Prospective studies with standardized IOP measurement, confirmed medication adherence, and prespecified assessment of ocular and systemic covariates are needed to determine whether concomitant limaprost use is reproducibly associated with the clinical response to latanoprost and to clarify any potential underlying mechanism.

## Acknowledgments

We are grateful to the Veterans Health Service Medical Center for providing the data used in this study.

## Author contributions

**Conceptualization:** Heewon Bae, Tae Gu Kang.

**Data curation:** Heewon Bae, Tae Gu Kang.

**Formal analysis:** Heewon Bae.

**Investigation:** Heewon Bae.

**Methodology:** Heewon Bae.

**Supervision:** Heewon Bae.

**Writing – original draft:** Heewon Bae.

**Writing – review & editing:** Heewon Bae, Tae Gu Kang.

**Validation:** Tae Gu Kang.

**Visualization:** Tae Gu Kang.
